# Characterization of an inorganic scintillator for small‐field dosimetry in MR‐guided radiotherapy

**DOI:** 10.1002/acm2.13012

**Published:** 2020-08-25

**Authors:** Davide Cusumano, Lorenzo Placidi, Emiliano D'Agostino, Luca Boldrini, Sebastiano Menna, Vincenzo Valentini, Marco De Spirito, Luigi Azario

**Affiliations:** ^1^ Fondazione Policlinico Universitario A. Gemelli IRCCS Rome Italy; ^2^ DoseVue NV Bioville Diepenbeek Belgium

**Keywords:** inorganic scintillator, MR‐guided radiotherapy, real‐time dosimetry, small‐field dosimetry

## Abstract

**Introduction:**

Aim of this study is to dosimetrically characterize a new inorganic scintillator designed for magnetic resonance‐guided radiotherapy (MRgRT) in the presence of 0.35 tesla magnetic field (B).

**Methods:**

The detector was characterized in terms of signal to noise ratio (SNR), reproducibility, dose linearity, angular response, and dependence by energy, field size, and B orientation using a 6 MV magnetic resonance (MR)‐Linac and a water tank.

Field size dependence was investigated by measuring the output factor (OF) at 1.5 cm. The results were compared with those measured using other detectors (ion chamber and synthetic diamond) and those calculated using a Monte Carlo (MC) algorithm.

Energy dependence was investigated by acquiring a percentage depth dose (PDD) curve at two field sizes (3.32 × 3.32 and 9.96 × 9.96 cm^2^) and repeating the OF measurements at 5 and 10 cm depths.

**Results:**

The mean SNR was 116.3 ± 0.6. Detector repeatability was within 1%, angular dependence was <2% and its response variation based on the orientation with respect to the B lines was <1%.

The detector has a temporal resolution of 10 Hz and it showed a linear response (R^2^ = 1) in the dose range investigated. All the OF values measured at 1.5 cm depth using the scintillator are in accordance within 1% with those measured with other detectors and are calculated using the MC algorithm. PDD values are in accordance with MC algorithm only for 3.32 × 3.32 cm^2^ field. Numerical models can be applied to compensate for energy dependence in case of larger fields.

**Conclusion:**

The inorganic scintillator in the present form can represent a valuable detector for small‐field dosimetry and periodic quality controls at MR‐Linacs such as dose stability, OFs, and dose linearity.

In particular, the detector can be effectively used for small‐field dosimetry at 1.5 cm depth and for PDD measurements if the field dimension of 3.32 × 3.32 cm^2^ is not exceeded.

## 1 INTRODUCTION

Magnetic resonance‐guided radiotherapy (MRgRT) represents to date one of the most promising techniques in the framework of personalized cancer care, offering high soft tissue contrast imaging and allowing precise radiation delivery.^1^, ^2^, ^3^

The hybrid machines designed for MRgRT combine linear accelerators with an onboard magnetic resonance (MR) scanner and differ in terms of main architecture of the system and magnetic field (B) strength.

The two systems currently available for clinical practice use a transverse geometry system where the B lines are perpendiculary oriented with respect to the radiation beam. Unity (Elekta, Stockholm, Sweden) combines a 1.5 T MR scanner with a 7 MV Flattening Filter Free (FFF) Linac, while MRIdian (ViewRay, Mountain View, California, United States) joins a 0.35 T MR scanner with 6 MV FFF Linac.^4^, ^5^, ^6^

The introduction of these hybrid machines has led to the necessity of new dosimetry systems for radiation beam quality controls, whose response would be constant in the presence of B and at the same time accurate for small‐field dosimetry.^7^

Furthermore, there is a growing interest in detectors able to provide a response characterized by high temporal resolution, to start exploring the possibility of in‐vivo applications in MRgRT.

The first detectors used for absolute dose measurements in the presence of B were ion chambers, for which a new formalism was introduced, including correction factors to take into consideration the dependence of the detector response from its orientation with respect to the B field lines.^8^, ^9^

Alternative systems for relative dosimetry were also tested, such as diamonds, diodes, and radiochromic films, with remarkable results in different experiences using MR‐Linac systems.^10^, ^11^, ^12^

Thanks to their physical properties, optical fiber‐based detectors appear to be particularly promising for applications of relative dosimetry in MRgRT, since they offer a real‐time response, are potentially accurate for small‐field dosimetry and are characterized by a light yield constant in the presence of a B with known strength.^13^

Even if the dosimetric properties of such detectors have been widely investigated for external beam radiotherapy (EBRT) and brachytherapy, limited experiences in MRgRT setting are today reported.^14^, ^15^, ^16^

The aim of this study was to dosimetrically characterize a new inorganic scintillator designed for MRgRT in the presence of 0.35 T B and to evaluate its clinical feasibility for small‐field dosimetry of MR‐Linac systems.

## 2 MATERIALS AND METHODS

### 2.A The detector

DoseWire Series 200 (DoseVue, N. V, Diepenbeek, Belgium) is an inorganic scintillator detector consisting of a hemisphere of 0.5 mm radius coupled to an optical fiber. The scintillating material is based on europium‐doped yttrium oxide and emits in the 600–650 nm window. This emission band helps increasing signal to noise ratio (SNR) thanks to the high scintillator light yield and the reduced presence of stem effect at these wavelengths.

The detector has a sensitive volume of 0.00026 cc and an effective point of measurement (EPOM) located at 3r/8, 'where r is the radius of the semi‐sphere. The scintillator has density of 3.4 g/cm3 and effective atomic number equal to 30.79.

The full system is designed as a 4‐channel device, allowing real‐time dose measurements at four locations simultaneously, with a maximum sampling frequency of 10 Hz.

The digital signal is then sent to a controlling computer, where a web‐based software interface displays the cumulative number of counts per channel.

**Fig. 1:**
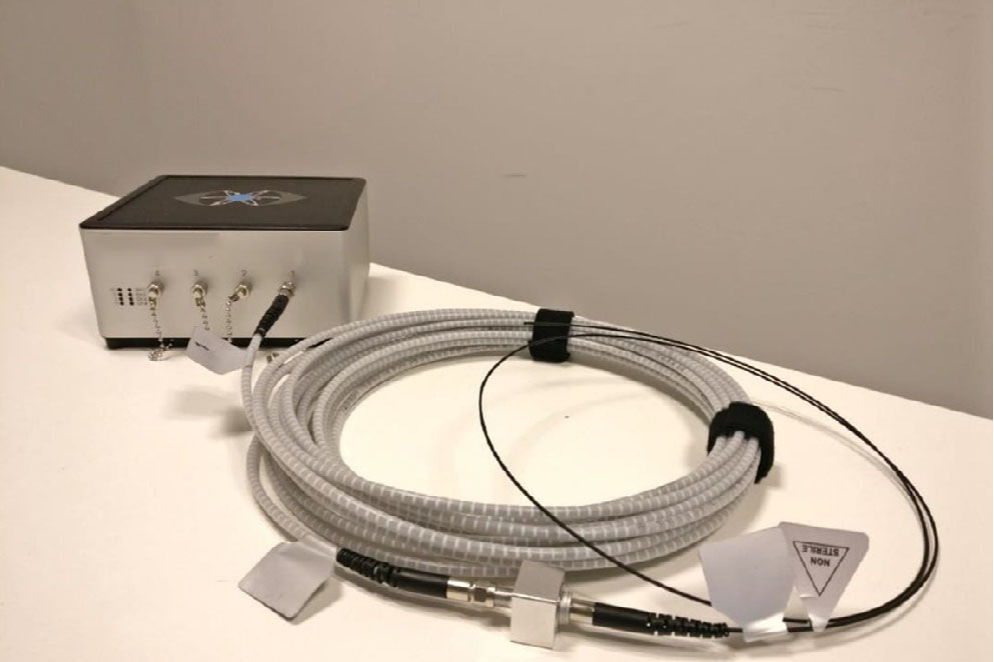
A picture of the Dose Wire Series 200 system.

Figure 1 shows the DoseWire Series 200 system, constituted by the inorganic scintillator, the optical fiber, and the detector reader.

The design of the detector (i.e., choice of inorganic scintillator) and the emission wavelength window are designed to minimize the stem signal.

Manufacturer data reported that the system has a response independent of dose rate (variation < 1% in a range of 600‐1400 MU/min) and a signal stable for cumulative dose up to 500 Gy (maximum variation equal to 0.6%). These data were confirmed in a recent experience performed on a standard linac.^17^

The system also shows good stability in terms of response with respect to the temperature variation, with variations within 1% in the range 17°–28°C.^18^ Such range can be extended up to 15–40°C, thanks to literature studies performed on detectors with similar scintillator composition that showed 1.3% variation between 15°C and 40°C.^19^

### 2.B Experimental measurements

The physical characterization of the detector was realized in the presence of 0.35 T B"/>, using a hybrid 6 MV low tesla MR‐Linac (MRIdian Linac, ViewRay, Mountain View, CA, US) and a manual water tank (PTW, Freiburg, Germany).

The detector has been characterized in terms of reproducibility, dose linearity, angular response, time‐dependent luminescence, and dependence by energy, field size, and B orientation.

**Fig. 2:**
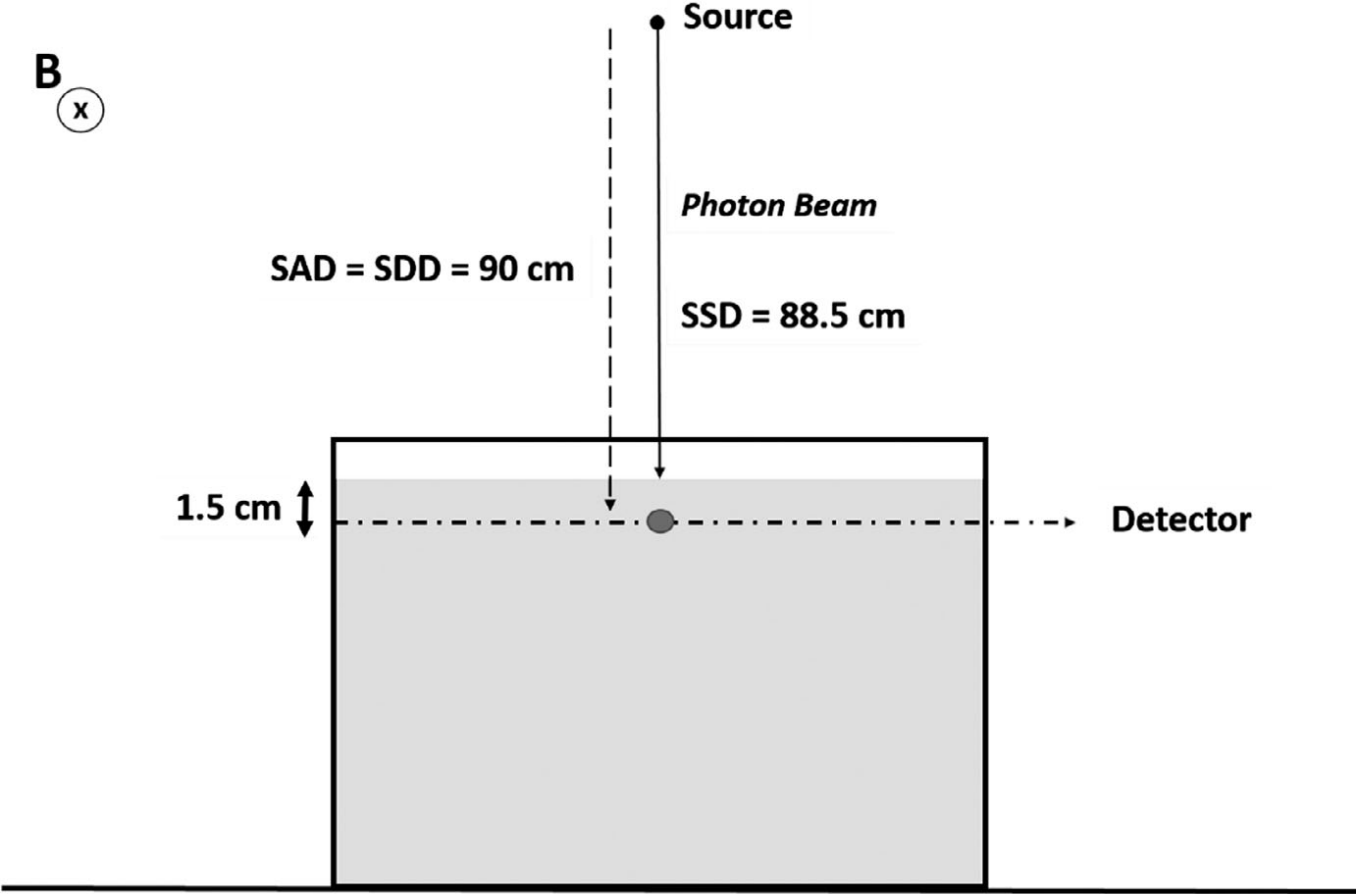
Schematic representation of the experimental setup adopted for the detector characterization. The magnetic field(B) lines are perpendicular to the plan of the figure.

A preliminary dose calibration was performed by exposing the detector to a 9.96 × 9.96 cm^2^ field and delivering 100 MU, adopting the experimental setup reported in Fig. 2.

The detector was placed in a water tank, at 1.5 cm depth. The source to axis distance (SAD) is 90 cm, the source to surface distance (SSD) was 88.5 cm and the source to detector distance (SDD) was the same as the SAD.

In this experimental setup, 1 MU corresponds to a dose value of 1 cGy according to the machine calibration that was performed following the TG‐51 protocol.^20^

A detector holder was realized in‐house, to ensure stable positioning of the detector. The MR‐Linac dose rate was 600 MU/min.

The number of counts generated by the scintillator was associated to the dose value measured by a 0.125 cc ion chamber (PTW 31010 Semiflex, Freiburg, Germany) placed in the same experimental conditions to calibrate the scintillator in dose values.

The detector was then characterized in terms of SNR to by varying the sampling rate, time‐dependent luminescence, response dependence from B orientation, angular dependence, reproducibility, and dose linearity with the same calibration experimental setup.

SNR was evaluated by delivering 100 MU thrice with a 9.96 × 9.96 cm^2^ field and repeating the measurements using a bare fiber (i.e., a fiber without scintillator detector) to estimate the noise level. SNR measurements were repeated at three different sampling rates: 2, 5, and 10 Hz.

Dose linearity was investigated delivering 1, 2, 5, 10, 20, 50, 60, 100, 200, 500, and 1000 MU and calculating the R^2^ correlation coefficient.

Time‐dependent luminescence was investigated using the experimental scheme proposed by Kertscher *et al*: the raw detector signal was acquired during two 5000 MU irradiations (corresponding to 50 Gy), with a time interval of 500 s between the two acquisitions.^21^ The raw signal variation was measured during both the irradiations: the mean signal within 1 s at 24 and 50 Gy was compared with the corresponding values after 1 Gy.

B orientation dependence was evaluated placing the detector parallel (0°) or perpendicular (90° and 270°) to the direction of B field lines and delivering three times 100 MU per configuration. A visual scheme of the different orientations is reported in Fig. S1.

Angular dependence was investigated by delivering 100 MU at different gantry angles (in a range from 270° to 90° in steps of 15 degrees) on the detector placed in a cylindrical phantom and oriented in parallel with respect to the B field lines. The photon beam remains perpendicular to the detector axis during the test, the gantry angles between 90° and 270° were not included in the analysis due to the presence of treatment couch.

Field size dependence was also investigated measuring the output factor (OF) under the same experimental conditions (detector placed at 1.5 cm depth).

The 9.96 × 9.96 cm^2^ field was considered as reference and different field sizes were investigated, always delivering 100 MU: 0.83 × 0.83 cm^2^, 1.66 × 1.66 cm^2^, 2.49 × 2.49 cm^2^, 3.32 × 3.32 cm^2^, 6.64 × 6.64 cm^2^, 8.30 × 8.30 cm^2^, 9.96 × 9.96 cm^2^, and 12.45 × 12.45 cm^2^). The non‐integer values of the field sizes are due to the MLC discretization of the MRIdian system.

The results were then compared with those measured using the 0.125 cc ion chamber (PTW 31010 Semiflex, Freiburg, Germany) and a 0.004 mm^3^ synthetic diamond (PTW 60019, Freiburg Germany) and those calculated using a Monte Carlo (MC) Treatment Planning System (TPS, version 4.5.1.239, ViewRay, Mountain View, California, USA) setting a dose calculation grid size of 1 mm and 2.4 million of histories. The small field correction factors proposed by TRS 483 (and reported in Supporting Information) were applied to the values measured using the ion chamber and the microdiamond.^22^

Energy dependence was investigated by repeating the scintillator OF measurements at two different depths (5 and 10 cm) and acquiring the Percentage Depth Dose (PDD) curve at two field size (3.32 × 3.32 cm^2^ and 9.96 × 9.96 cm^2^).

PDD curves were acquired at SSD equal to 78 cm, using the scintillator and the ion chamber and comparing the results with those calculated using the MC TPS. The following depths were considered: 10, 15, 20, 30, 50, 70, 100, and 150 mm.

## 3 RESULTS

**Fig. 3:**
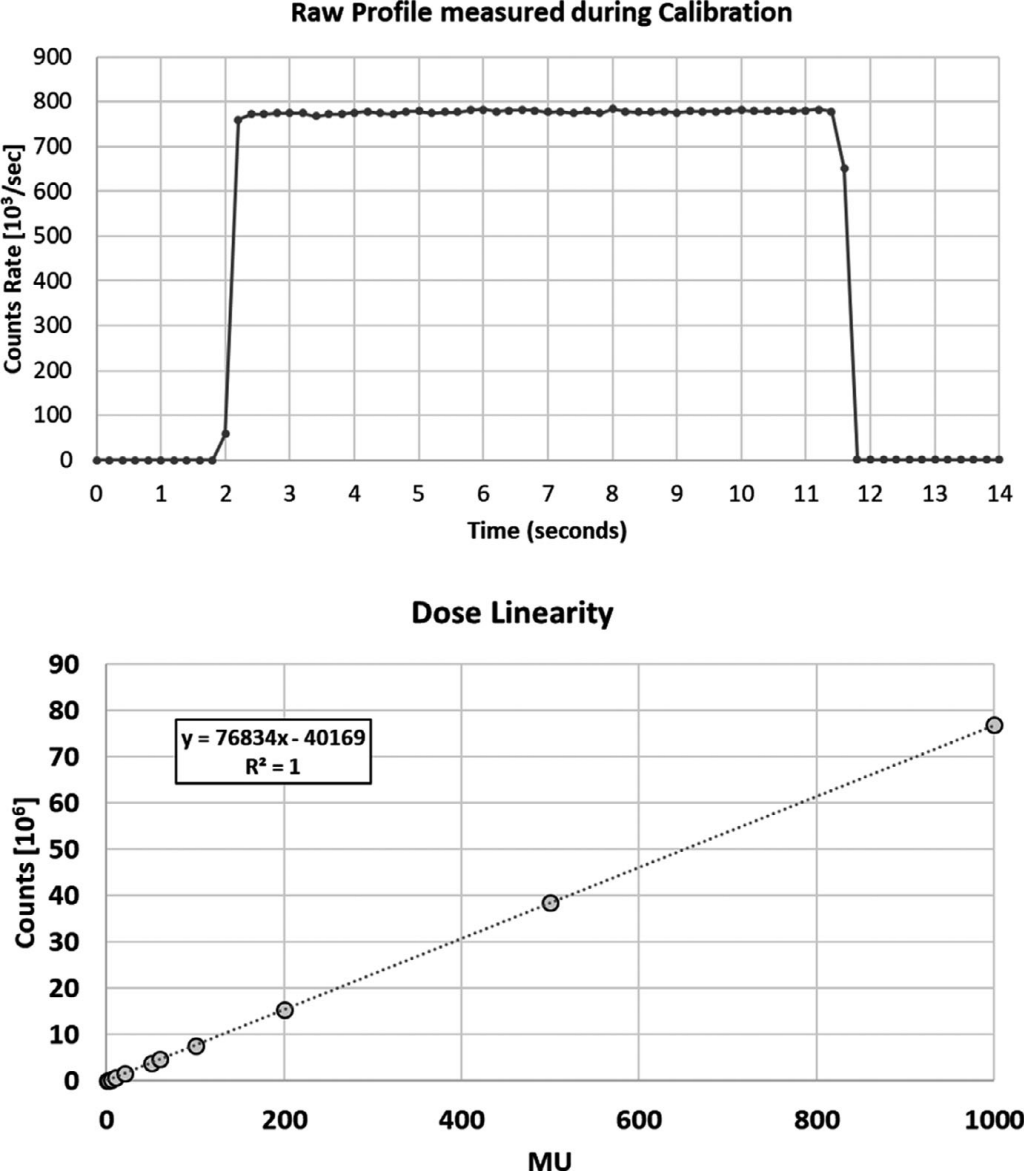
Raw Profile acquired with the inorganic scintillator during calibration in function of time (top) and scintillator response to variations in the monitor units (bottom).

Figure 3 shows the detector raw signal as a function of time obtained during calibration and the scintillator response to variations of the MU, once the detector is calibrated.

The SNR was equal to 116.2 ± 0.6 at 1 Hz, 116.3 ± 0.6 at 5 Hz, and 116.1 ± 0.7 at 10 Hz. The whole raw data are reported in Table S1.

The response variation with varying sampling rates is within 0.5%. A temporal resolution of 10 Hz was chosen for all the measurements considering the delivery of more than 10 MU.

In the graph of the dose linearity, the error bars did not exceed the box sizes. The R^2^ correlation coefficient was equal to 0.999.

As regards time‐dependent luminescence, Fig. S2 reports the raw signal acquired in function of time for the two 5000 MU acquisitions. With respect to the signal intensity registered after 1 Gy, an increase in scintillation was observed in both the irradiations equal to 0.3%/0.6% after 24 Gy and 0.5%/0.8% after 50 Gy.

The response dependence with respect to the orientation of the detector in the B lines is shown in Table 1.

**Table 1:** Variability of detector response *when changing its orientation with respect to the magnetic field (B) force lines*.

	Detector orientation with respect to B		
	0	90	270
Measurement 1 (counts)	8,11E+06	8,05E+06	8,04E+06
Measurement 2 (counts)	8,11E+06	8,05E+06	8,06E+06
Measurement 3 (counts)	8,13E+06	8,07E+06	8,09E+06
Mean value (counts)	8,12E+06	8,06E+06	8,08E+06
Standard deviation	1,26E+03	1,37E+03	3,04E+03
Coefficient of variation	0,16%	0,29%	0,37%
Mean dose (Gy)	1,00	0,993	0,995

**Fig. 4:**
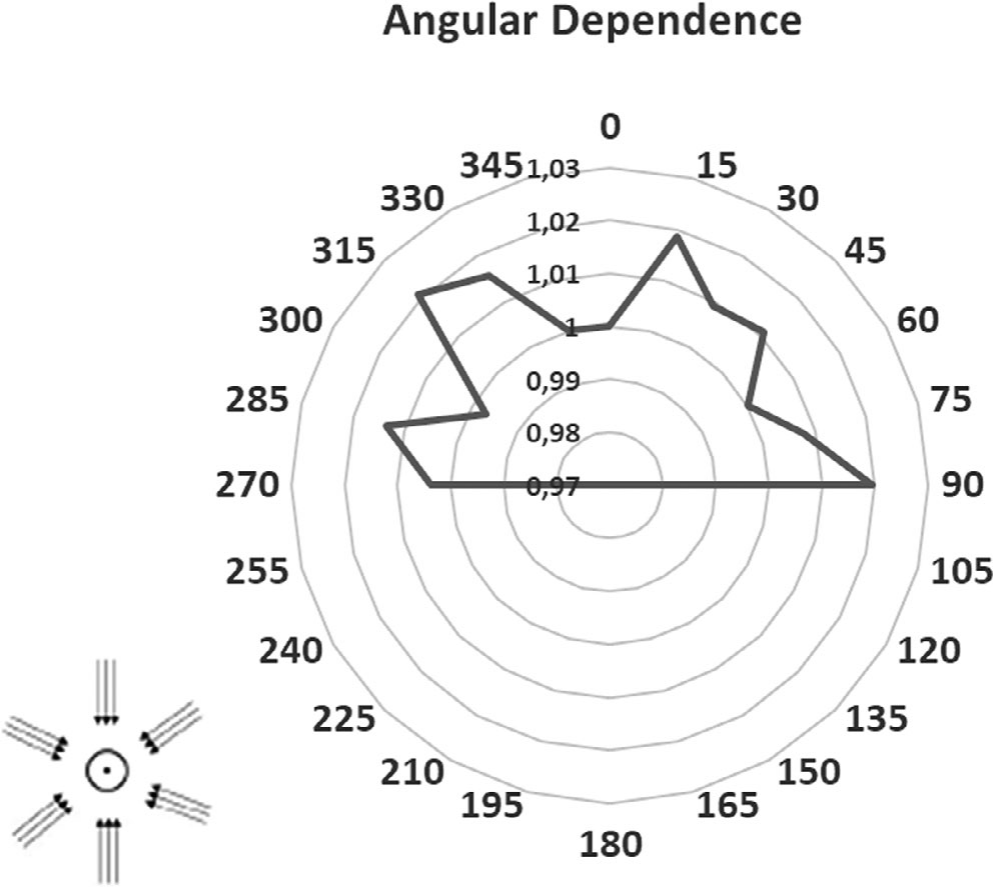
Angular dependence along the detector axis. Measurements were performed from 270° to 90° by steps of 15°. A visual representation of the detector orientation with respect to the beam axis is reported at the bottom — left of the figure.

The detector signal repeatability was below 0.4%, as expressed by the coefficient of variation. The response variation when changing the detector orientation with respect to the B lines, was below 1% (−0.7% at 90° and −0.5% at 270°). The measurements related to the angular dependence of the detector are reported in Fig. 4, together with a visual representation of the detector orientation with respect to the beam axis.

**Fig. 5:**
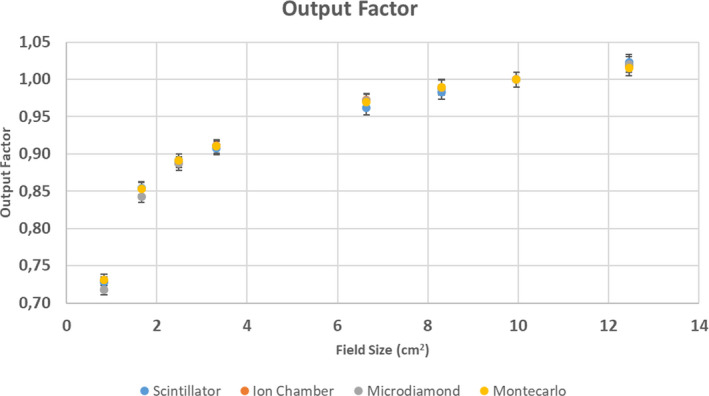
Output factors measured with inorganic scintillator, ion chamber, microdiamond, and calculated with Monte Carlo treatment planning system (TPS).

The angular response of the detector is within 2% for all the gantry angles considered. Figure 5 reports the OF values measured at 1.5 cm depths using the scintillator, the ion chamber, the microdiamond, and calculated with MC simulation. The OF measured using the ion chamber is not available for field sizes smaller than 2.49 cm.

All the OFs measured at 1.5 cm depth using the scintillator are in accordance within 1% with those measured using the other detectors and calculated by MC simulation.

For small fields (FS ≤ 2.49 cm), the values measured by the scintillator are the closest to those calculated by the TPS. Maximum variation in the OF measured at 1.5 cm depth using the scintillator is 0.8%, observed for 12.45 and 6.64 cm^2^ fields.

The OF measurements at 5 and 10 cm depths are reported in supplementary materials, together with the percentage difference with respect to the calculated values using TPS. The percentage error at these depths is higher (mean percentage error equal to 4,57% and −4,19% for 5 and 10 cm, respectively) and it can be compensated using appropriate correction factors, reaching values of 0.38% for 5 cm depth and −1.74% for 10 cm depth. The details related to the correction factors are reported in a dedicated section of the Supporting Information.

**Fig. 6:**
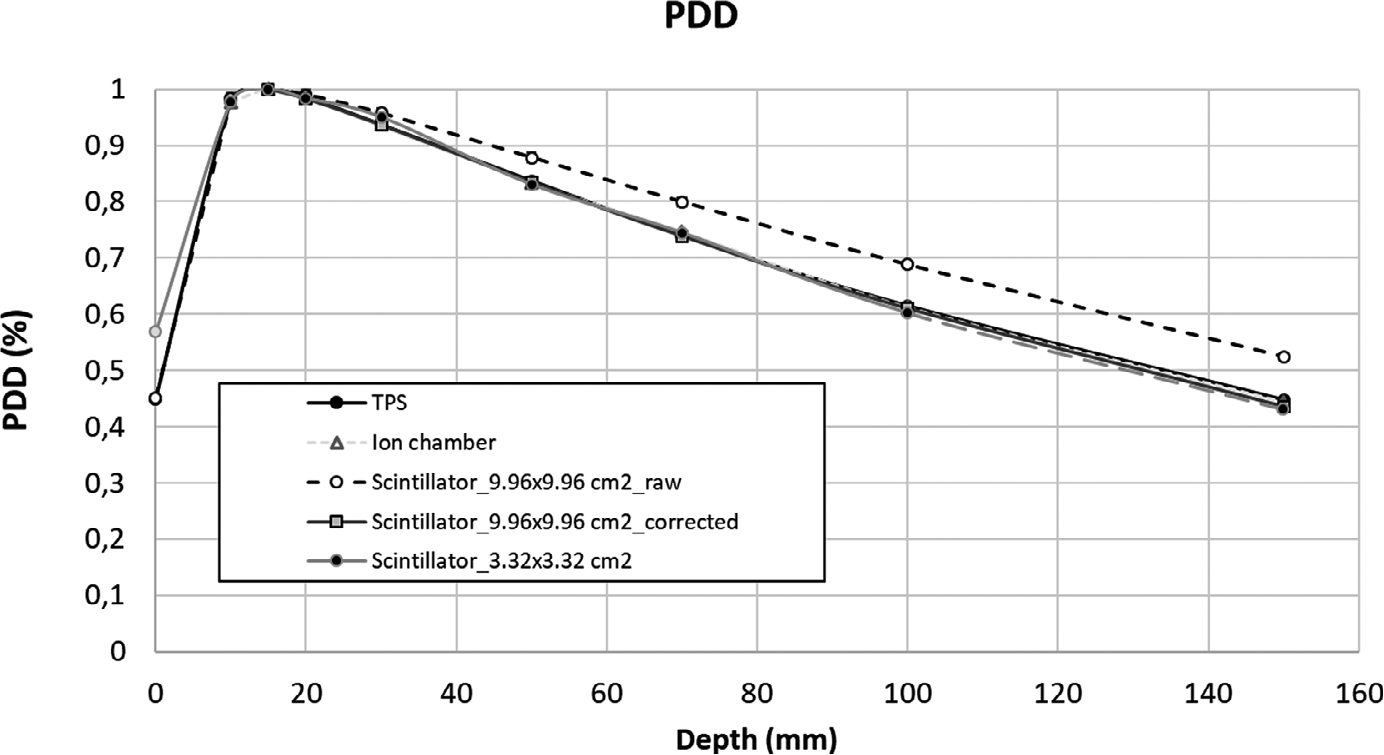
Percentage depth dose measured using the scintillator at two different field sizes and calculated using the Monte Carlo treatment planning system (TPS).

Figure 6 reports the PDD curves measured using the ion chamber and the scintillator at the two different field sizes and those calculated using the MC TPS. Error bars did not exceed the box sizes.

The PDD values measured with the ion chamber are in accordance with those calculated by the TPS within 1% for all the analyzed depth values (maximum difference was 0.9% at 150 mm depth).

The PPD values measured using the scintillator are in agreement with the TPS within 1% only for the 3.32 × 3.32 cm^2^ field. Regarding the 9.96 × 9.96 cm^2^ field, scintillator measurements are in accordance with TPS values within 1% of difference only for depth values lower than 2 cm. For larger depth, the disagreement linearly grew, reaching a maximum of 17% at depth equal to 15 cm. A numerical model was applied to compensate for the energy dependence in the case of 9.96 × 9.96 cm^2^ field. All the values measured and calculated are reported in supplementary materials.

## 4 DISCUSSION

In this work, the physical characterization of a new inorganic scintillator designed for small‐field dosimetry in MRgRT has been successfully performed.

Most of the fiber‐based detectors tested for EBRT and brachytherapy are organic scintillators, with very good results reported in terms of energy independence and dose accuracy.

The dose measurements performed using these systems were found to be in agreement with ion chamber measurements within 1% for 6–25 MV photon beams and for 8–21 MeV electron beams.^23^, ^24^, ^25^, ^26^

Some recent experiences have investigated the properties of organic scintillators in the presence of low‐ and high‐tesla B, concluding that these detectors can be effectively used for OF measurements in MRgRT.^27^, ^28^

However, plastic scintillators emit in the blue spectral region (400–450 nm) and their signal is usually heavily contaminated by the stem effect, due to the Cerenkov radiation and the fluorescence light induced in the optical fiber during the irradiation.^29^, ^30^ Several techniques were developed to reduce the stem effect, mainly focused on the use of an additional background fiber to estimate and suppress the Cerenkov radiation.^31^, ^32^

The recent interest toward the use of inorganic scintillators is justified by the fact that these materials show higher light yield, emitting in a spectral region where the aforementioned contaminating effects are significantly less prominent.^33^

The results of this study confirm that the inorganic scintillators show high SNR independent of the sampling rate chosen and they are characterized by a highly reproducible and linear response which is linear with the dose.

Time luminescence variation is below 1%, reporting good response for high dose values. The detector response is not influenced by the orientation of B lines (response variation < 1% for the different orientations investigated of the detector with respect to the B field lines) or by the beam’s orientation, reporting angular dependence within 2%.

Due to the reduced dimension of the detector, an eventual shift of the EPOM due to the B presence can be considered as negligible. To our knowledge, this represents the first example of inorganic scintillator use in the presence of B.

Based on the results obtained, the present detector shows response properties that make it useful for small‐field dosimetry and for many of the periodic quality controls necessary to the stability of a MR‐Linac system.

One of the drawbacks of this detector is today represented by the energy dependence: due to its high effective Z material, the detector tends to overestimate the dose contribution from low energy secondary radiation, reporting higher disagreement with respect to the TPS values when OF measurements are performed at depths larger than 1.5 cm or PDD curves are acquired at field sizes larger than 3.32 × 3.32 cm^2^.

Several strategies are currently under investigation to physically solve this dependence, such as the use of pass‐band filters or the physical changes in the fiber architecture. Pending these technological solutions, energy dependence can be compensated through the application of numerical models, such as those proposed in this study and detailed in Supporting Information.

Energy dependence today represents a bottleneck toward the in‐vivo application, together with the need of multiple simultaneous measurements: different research projects are currently under investigation to implement such scintillators in a multi‐channel system, mandatory for the verification of an intensity‐modulated dose distribution.

## 5 CONCLUSION

The basic physical characterization of a new inorganic scintillator designed for MRgRT application has been successfully performed.

In the present form, this inorganic scintillator can represent a valuable tool for quality controls and relative dosimetry on MR‐Linacs, such as dose stability during the different treatment days, OFs, and dose linearity check.

In particular, the detector can be effectively used for small‐field dosimetry at 1.5 cm depth and for PDD measurements if the field dimension of 3.32 × 3.32 cm^2^ is not exceeded.

Thanks to its high temporal resolution and its independence of B orientation, this system, if properly compensated for the energy dependence and integrated into two‐dimensional arrays, can also represent a promising solution of in‐vivo dose measurements, strongly required in MRgRT applications.

## CONFLICT OF INTERESTS

Dr. Cusumano, Boldrini, and Placidi received speaker honoraria from an agreement with ViewRay, outside the present work. Prof. Valentini has a research agreement with ViewRay, outside the present work. All the other authors have no conflict of interests to declare.

## Supporting information


**Fig. S1.** Detector orientation with respect the magnetic field force lines. The scheme reports in axial view the treatment couch (grey) and the magnetic field force lines. In blue is represented the water tank, in yellow the radiation field. The black box at the corner of the figure represents the photomultiplier of the DoseWire system. The detector direction is represented in red color. In the measurements performed the detector was oriented at 0° (left), 90° (center) or 270° (right) with respect to the B force lines.
**Fig. S2** **.** Results of raw signal acquired for time luminescence measurements.

**Table S1.** SNR in function of sampling rate.
**Table S2** **.** Small Field correction factors used for ion chamber (Semiflex PTW 31010) and Microdiamond (PTW 60019) as reported in TRS 483.
**Table S3** **.** OF values calculated using the Montecarlo (TPS) and measured using the scintillator (SC), the ion chamber and the microdiamond at 1,5 depth. In table are present not only the raw values but also those corrected using the correction factors for ion chamber (IC) and microdiamond (MD). The percentage difference between the measured values and the calculated ones are also reported. No correction factors are applied for scintillator.
**Table S4** **.** OF values calculated using the TPS and measured using the scintillator at 5 depth. In table are present not only the raw values but also those corrected using the numerical model. The percentage difference between the measured values and the calculated ones are also reported.
**Table S5** **.** OF values calculated using the TPS and measured using the scintillator at 10 depth. In table are present not only the raw values but also those corrected using the numerical model. The percentage difference between the measured values and the calculated ones are also reported.
**Table S6** **.** Experimental measurements for PDD acquisition and percentage difference with respect TPS.

**Data S1.** Correction factors proposed for OF at 5 cm and 10 cm depths.
**Data S2.** Correction factors proposed for PDD measurement at 10x10 cm^2^
