# Supplementary material for: Characterization of an inorganic scintillator for small‐field dosimetry in MR‐guided radiotherapy
Source: J Appl Clin Med Phys. 2020 Aug 25;21(9):244–51. doi: 10.1002/acm2.13012 (PMC7497936; doi:10.1002/acm2.13012)

**Supplementary materials**

Figure 1 - Detector orientation with respect the magnetic field force lines. The scheme reports in axial view the treatment couch (grey) and the magnetic field force lines. In blue is represented the water tank, in yellow the radiation field. The black box at the corner of the figure represents the photomultiplier of the DoseWire system. The detector direction is represented in red color. In the measurements performed the detector was oriented at 0° (left), 90° (center) or 270° (right) with respect to the B force lines


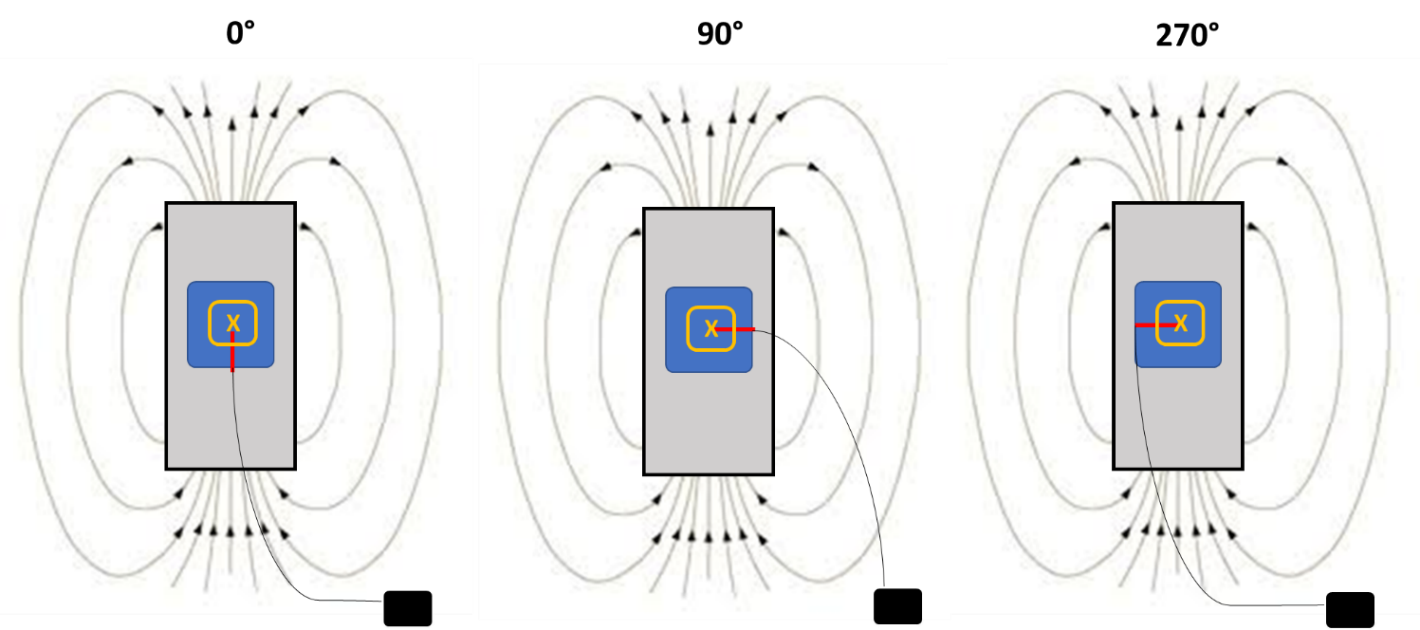


Figure 2 – Results of raw signal acquired for time luminescence measurements


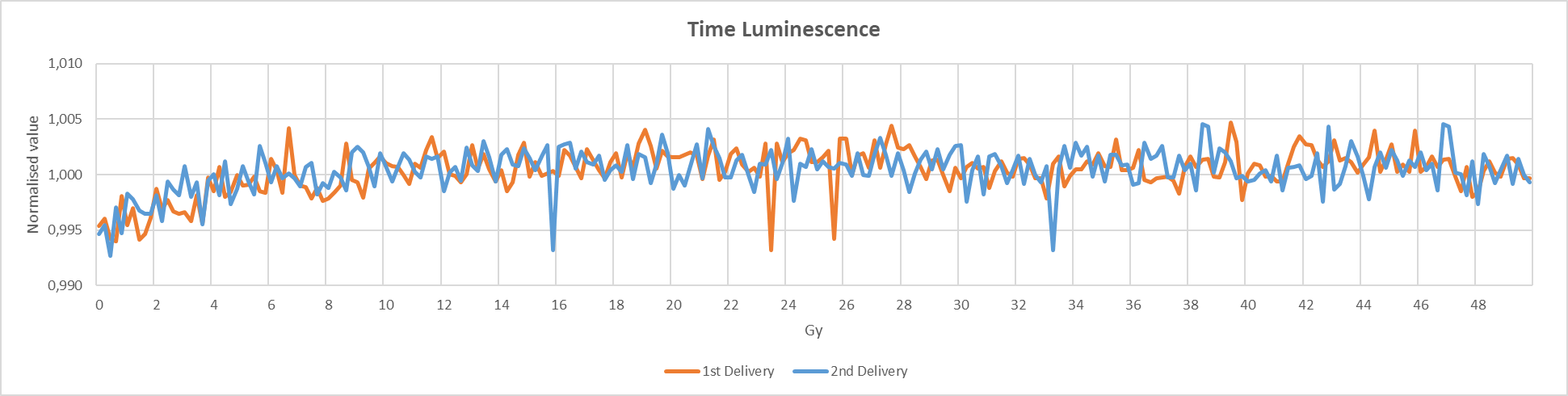

Supplement: Supplementary file 1 — Fig. S1. Detector orientation with respect the magnetic field force lines. The scheme reports in axial view the treatment couch (grey) and the magnetic field force lines. In blue is represented the water tank, in yellow the radiation field. The black box at the corner of the figure represents the photomultiplier of the DoseWire system. The detector direction is represented in red color. In the measurements performed the detector was oriented at 0° (left), 90° (center) or 270° (right) with respect to the B force lines. Fig. S2 . Results of raw signal acquired for time luminescence measurements. [file ACM2-21-244-s001.docx]
