# Supplementary material for: Characterization of an inorganic scintillator for small‐field dosimetry in MR‐guided radiotherapy
Source: J Appl Clin Med Phys. 2020 Aug 25;21(9):244–51. doi: 10.1002/acm2.13012 (PMC7497936; doi:10.1002/acm2.13012)
Supplement: Supplementary file 2 — Table S1. SNR in function of sampling rate. Table S2 . Small Field correction factors used for ion chamber (Semiflex PTW 31010) and Microdiamond (PTW 60019) as reported in TRS 483. Table S3 . OF values calculated using the Montecarlo (TPS) and measured using the scintillator (SC), the ion chamber and the microdiamond at 1,5 depth. In table are present not only the raw values but also those corrected using the correction factors for ion chamber (IC) and microdiamond (MD). The percentage difference between the measured values and the calculated ones are also reported. No correction factors are applied for scintillator. Table S4 . OF values calculated using the TPS and measured using the scintillator at 5 depth. In table are present not only the raw values but also those corrected using the numerical model. The percentage difference between the measured values and the calculated ones are also reported. Table S5 . OF values calculated using the TPS and measured using the scintillator at 10 depth. In table are present not only the raw values but also those corrected using the numerical model. The percentage difference between the measured values and the calculated ones are also reported. Table S6 . Experimental measurements for PDD acquisition and percentage difference with respect TPS. [file ACM2-21-244-s002.docx]

**Supplementary materials**

Table 1 – SNR in function of sampling rate

| **Frequency** | **Signal/Noise** | **Meas_1** | **Meas_2** | **Meas_3** | **SNR_1** | **SNR_2** | **SNR_3** | **SNR** | **Dev.St** |
| --- | --- | --- | --- | --- | --- | --- | --- | --- | --- |
| 1 Hz | Signal | 922202 | 922531 | 925651 | 115,6 | 116,8 | 116,3 | 116,2 | 0,6 |
| 1 Hz | Noise | 7980 | 7898 | 7960 |  |  |  |  |  |
| 5 Hz | Signal | 926766 | 923232 | 927552 | 116,8 | 115,7 | 116,3 | 116,3 | 0,6 |
| 5 Hz | Noise | 7932 | 7978 | 7978 |  |  |  |  |  |
| 10 Hz | Signal | 938454 | 923685 | 928924 | 116,5 | 115,3 | 116,6 | 116,1 | 0,7 |
| 10 Hz | Noise | 8056 | 8012 | 7965 |  |  |  |  |  |

Table 2 – Small Field correction factors used for ion chamber (Semiflex PTW 31010) and Microdiamond (PTW 60019) as reported in TRS 483

| ***Field Size (cm2)*** | ***Ion Chamber*** | ***Microdiamond*** |
| --- | --- | --- |
| 0,83 |  | 0,977 |
| 1,66 |  | 0,993 |
| 2,49 | 1,002 | 0,999 |
| 3,32 | 1,001 | 1,000 |
| 6,64 | 1,000 | 1,000 |
| 8,30 | 1,000 | 1,000 |
| 9,96 | 1,000 | 1,000 |
| 12,45 | 1,000 | 1,000 |

Table 3 – OF values calculated using the Montecarlo (TPS) and measured using the scintillator (SC), the ion chamber and the microdiamond at 1,5 depth. In table are present not only the raw values but also those corrected using the correction factors for ion chamber (IC) and microdiamond (MD). The percentage difference between the measured values and the calculated ones are also reported. No correction factors are applied for scintillator

| **Field size (cm)** | **TPS** | **Raw Measurements** | | | **Corrected Values** | | **Percentage Difference with TPS** | | |
| --- | --- | --- | --- | --- | --- | --- | --- | --- | --- |
|  |  | **SC** | **IC** | **MD** | **IC** | **MD** | **SC** | **IC** | **MD** |
| 0,83 | 0,73 | 0,73 |  | 0,74 |  | 0,72 | -0,4% |  | 0,5% |
| 1,66 | 0,85 | 0,85 |  | 0,85 |  | 0,84 | 0,1% |  | -0,5% |
| 2,49 | 0,89 | 0,89 | 0,89 | 0,89 | 0,89 | 0,89 | 0,0% | -0,4% | -0,3% |
| 3,32 | 0,91 | 0,91 | 0,91 | 0,91 | 0,91 | 0,91 | -0,2% | 0,1% | 0,0% |
| 6,64 | 0,97 | 0,96 | 0,97 | 0,97 | 0,97 | 0,97 | -0,8% | 0,2% | 0,1% |
| 8,30 | 0,99 | 0,98 | 0,99 | 0,99 | 0,99 | 0,99 | -0,7% | -0,1% | -0,1% |
| 9,96 | 1,00 | 1,00 | 1,00 | 1,00 | 1,00 | 1,00 | 0,0% | 0,0% | 0,0% |
| 12,45 | 1,02 | 1,02 | 1,02 | 1,02 | 1,02 | 1,02 | 0,8% | 0,3% | 0,5% |

Table 4 – OF values calculated using the TPS and measured using the scintillator at 5 depth. In table are present not only the raw values but also those corrected using the numerical model. The percentage difference between the measured values and the calculated ones are also reported.

| **Field Size (cm)** | **Values at 5 cm depth** | | | **Percentage Difference** | |
| --- | --- | --- | --- | --- | --- |
|  | ***Montecarlo*** | **SC_raw** | **SC_Corr** | **SC_raw** | **SC_Corr** |
| 0,83 | 0,73 | 0,65 | 0,67 | -10,87% | -8,59% |
| 1,66 | 0,85 | 0,78 | 0,85 | -7,77% | 0,17% |
| 2,49 | 0,89 | 0,83 | 0,89 | -6,59% | -0,32% |
| 3,32 | 0,91 | 0,86 | 0,91 | -5,38% | 0,36% |
| 6,64 | 0,97 | 0,95 | 0,97 | -2,12% | -0,45% |
| 8,3 | 0,99 | 0,98 | 0,98 | -0,56% | -0,95% |
| 9,96 | 1,00 | 1,00 | 1,00 | 0,00% | 0,00% |
| 12,45 | 1,02 | 1,06 | 0,99 | 3,95% | -2,40% |

Table 5 – OF values calculated using the TPS and measured using the scintillator at 10 depth. In table are present not only the raw values but also those corrected using the numerical model. The percentage difference between the measured values and the calculated ones are also reported.

| **Field Size (cm)** | **Values at 10 cm depth** | | | **Percentage Difference** | |
| --- | --- | --- | --- | --- | --- |
|  | ***Montecarlo*** | **SC_raw** | **SC_Corr** | **SC_raw** | **SC_Corr** |
| 0,83 | 0,57 | 0,54 | 0,59 | -4,48% | 4,24% |
| 1,66 | 0,77 | 0,71 | 0,77 | -8,00% | -0,39% |
| 2,49 | 0,82 | 0,75 | 0,81 | -7,93% | -1,20% |
| 3,32 | 0,85 | 0,79 | 0,84 | -7,17% | -1,05% |
| 6,64 | 0,93 | 0,90 | 0,93 | -3,23% | -0,14% |
| 8,3 | 0,96 | 0,94 | 0,96 | -1,72% | 0,02% |
| 9,96 | 1,00 | 1,00 | 1,00 | 0,00% | 0,00% |
| 12,45 | 1,01 | 1,04 | 1,02 | 2,99% | 0,70% |

Table 6 – Experimental measurements for PDD acquisition and percentage difference with respect TPS

| **Depth**  **(mm)** | **TPS** | **Measured Values** | | | | **Percentage Difference with respect TPS** | | | |
| --- | --- | --- | --- | --- | --- | --- | --- | --- | --- |
|  |  | **Ion Chamber** | **10_raw** | **10_cor** | **3_raw** | **Ion Chamber** | **10_raw** | **10_cor** | **3_raw** |
| 10 | 0,979 | 0,98 | 0,983 | 0,980 | 0,977 | -0,28 | 0,42 | 0,10 | -0,16 |
| 15 | 1 | 1 | 1 | 1 | 1 | 0,00 | 0,00 | 0,00 | 0,00 |
| 20 | 0,986 | 0,98 | 0,990 | 0,983 | 0,984 | -0,16 | 0,44 | -0,31 | -0,25 |
| 50 | 0,837 | 0,83 | 0,878 | 0,834 | 0,830 | -0,37 | 4,93 | -0,30 | -0,80 |
| 70 | 0,74 | 0,75 | 0,800 | 0,739 | 0,744 | 0,67 | 7,90 | -0,33 | 0,45 |
| 100 | 0,615 | 0,61 | 0,688 | 0,610 | 0,610 | -0,43 | 11,85 | -0,80 | -0,81 |
| 150 | 0,448 | 0,44 | 0,524 | 0,436 | 0,450 | -0,90 | 16,97 | -2,73 | 0,45 |
