# Supplementary material for: Characterization of an inorganic scintillator for small‐field dosimetry in MR‐guided radiotherapy
Source: J Appl Clin Med Phys. 2020 Aug 25;21(9):244–51. doi: 10.1002/acm2.13012 (PMC7497936; doi:10.1002/acm2.13012)
Supplement: Supplementary file 3 — Data S1. Correction factors proposed for OF at 5 cm and 10 cm depths. Data S2. Correction factors proposed for PDD measurement at 10x10 cm2 [file ACM2-21-244-s003.docx]

**Supplementary materials**

**Correction factors proposed for OF at 5 cm and 10 cm depths**

Considering *c_raw* the number of counts measured by the scintillator during the measurement i performed delivering a beam of field size equal to FS, the number of counts corrected is equal to:

$$c_{corr}(i)=\frac{c_{raw}(i)}{1+CF \frac{HFS(i)}{HFS(ref)}}$$

Where:

HFS (i) is the value of half field size in cm for the measurement i

HFS (ref) is the value of half field size in cm for the reference field (9.96 cm)

CF is the correction factor, equal to 0.25 for 5 cm depth and 0.1 for 10 cm depth.

**Correction factors proposed for PDD measurement at 10x10 cm^2^**

Considering *c_raw* the number of counts measured by the scintillator during the measurement performed at depth d delivering a 0.96x9.96 cm^2^ beam, the number of counts corrected is equal to:

$$c_{corr}(d)=\frac{c_{raw}(d)}{1+CF \left( d-d_{max} \right)}$$

Where:

d_max_ is the build-up depth, equal to 1.5 cm

CF is the correction factor, equal to 0.0015
